# Feedbackgespräche in der Weiterbildung: Strukturiertes Feedback in der anästhesiologischen Weiterbildung am Universitätsklinikum Hamburg-Eppendorf – eine Prä-Post-Analyse

**DOI:** 10.1007/s00101-026-01700-3

**Published:** 2026-06-24

**Authors:** Nicolas Hoffmann, Martin Klasen, Katharina Röher, Saša Sopka

**Affiliations:** 1 Klinik für Anästhesiologie und Intensivmedizin, Albertinen-Krankenhaus, Hamburg, Deutschland; 2 Klinik und Poliklinik für Anästhesiologie, Universitätsklinikum Hamburg-Eppendorf, Hamburg, Deutschland; 3 AIXTRA Kompetenzzentrum für Training und Patientensicherheit – RWTH Aachen, Aachen, Deutschland; 4 Klinik für Anästhesiologie, Universitätsklinikum Aachen, Aachen, Deutschland

**Keywords:** Feedback, Medizinische Ausbildung, Arbeitsplatzrotation, Mitarbeitergespräch, Anästhesiologie, Job satisfaction, Workplace rotation, Medical education, Performance review, Anesthesiology

## Abstract

**Hintergrund:**

Feedback stellt einen der größten Stimuli für einen effektiven Lernfortschritt dar. Dennoch erhalten Weiterbildungsassistent:innen (WBA) in der klinischen Ausbildung zu wenig davon. Aktuell verpflichtende Weiterbildungsgespräche (WBG) werden unter den WBA als unzureichend wahrgenommen.

**Ziel der Arbeit:**

Um klinisches Feedback häufiger verfügbar zu machen, wurden 2018 in der Klinik für Anästhesiologie am Universitätsklinikum Hamburg-Eppendorf (UKE) strukturierte, klinische Feedbackgespräche (FBG) in der Weiterbildung begleitend zu den klinischen Arbeitsplatzrotationen eingeführt.

**Methoden:**

Der Effekt der Einführung dieser FBG wurde evaluiert, in dem vorher und nachher unter den WBA eine Umfrage zu Feedback in der Klinik und allgemeiner Zufriedenheit am Arbeitsplatz durchgeführt wurde.

**Ergebnisse:**

Initial beurteilten 64 %, dass die WBG eher nicht als Feedbackinstrument ausreichen. Nach Einführung der FBG wurden diese dagegen von 84 % eher als sinnvolles Instrument angesehen. Die WBA hatten vermehrt den Eindruck, sie wüssten, wie ihre Vorgesetzten ihre praktischen Fähigkeiten einschätzten (*p* = 0,017), würden von ihren Vorgesetzten so behandelt, wie sie es sich wünschten (*p* = 0,039), und könnten ihren Vorgesetzten eher ihre eigenen Kritikpunkte und Verbesserungsvorschläge problemlos vortragen (*p* = 0,033).

**Diskussion:**

Im Gegensatz zu den einmaligen WBG werden mehrmalige FBG pro Jahr von den WBA überwiegend begrüßt und als Lehrinstrument für das Verfügbarmachen von Feedback anerkannt. Diese FBG schaffen zusätzlich ein Forum, in dem WBA leichter Rückmeldungen an Vorgesetzte geben können. Damit scheinen FBG einen Beitrag für eine Feedbackkultur und wertschätzendes und respektvolles Arbeitsklima zu leisten.

**Graphic abstract:**

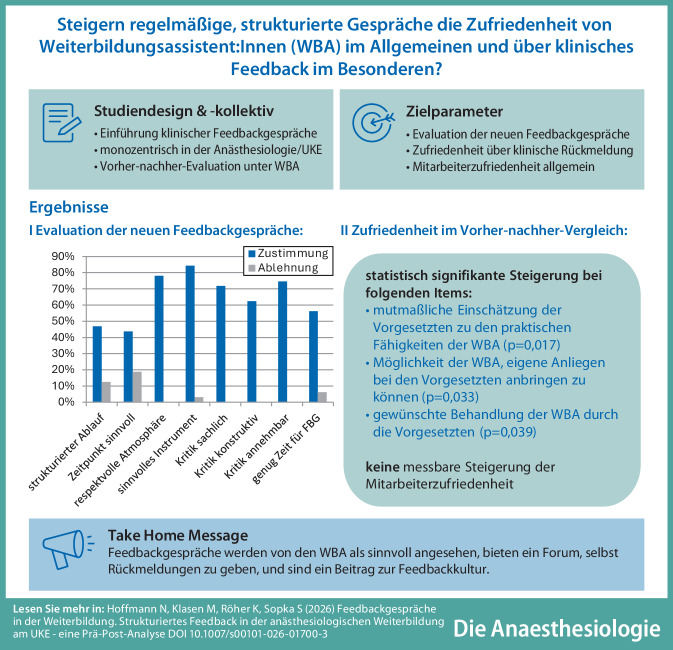

**Zusatzmaterial online:**

Die Online-Version dieses Beitrags (10.1007/s00101-026-01700-3) enthält die im Text erwähnten Anhänge A–C. Bitte scannen Sie den QR-Code.

## Abstract (en)

**Background:**

Feedback is one of the most powerful influences on learning and achievement. Nevertheless, residents receive too little of it during postgraduate clinical education. Currently mandatory annual professional development reviews are perceived as inadequate by residents.

**Objective:**

In order to make clinical feedback more frequently available, structured clinical feedback conversations were introduced in 2018 in the Department of Anesthesiology at the University Medical Center Hamburg-Eppendorf (UKE) in postgraduate education to accompany clinical workplace rotations.

**Methods:**

The effect of the introduction of these feedback conversations was evaluated by conducting a survey among residents before (time t_0_) and after (time t_1_) the introduction regarding general perceptions of received feedback, any desire for more clinical feedback and general job satisfaction. The research question was if the introduction of feedback conversations could improve satisfaction of received feedback on job performance and overall job satisfaction among the residents.

**Results:**

Approximately half of the residents (t_0_ 51%; t_1_ 44%) participated in the surveys. At t_0_ 64% initially judged the annual professional development reviews to be inadequate as a feedback tool. At t_1_ on average, 3.4 feedback conversations (mean 2) took place per resident, each lasting 10.4 min (mean 10 min). After the introduction of the feedback conversations, however, 84% viewed it as a more useful tool and 78% felt that the feedback conversations took place in an appreciative and respectful atmosphere. In comparison before and after the introduction of the feedback conversations the residents increasingly felt they knew how their supervisors assessed their practical skills (*p* = 0.017), were treated by their supervisors as they desired (*p* = 0.039) and were more likely to be able to easily present their own criticisms and suggestions for improvement (*p* = 0.033); however, no improvement could be observed in general job satisfaction.

**Conclusion:**

In contrast to the mandatory annual professional development reviews, multiple feedback conversations per year are generally welcomed by the residents and recognized as a teaching tool for providing feedback. These feedback conversations also create a forum in which residents can more easily provide feedback to superiors. Thus, feedback conversations appear to contribute to a feedback culture and an appreciative and respectful working environment.

Feedback stellt einen der größten Lernstimuli dar. Dennoch erhalten Weiterbildungsassistent:innen (WBA) in der klinischen Ausbildung zu wenig davon. Die in den aktuellen Weiterbildungsordnungen verpflichtenden Mitarbeitergespräche werden als unzureichend wahrgenommen. Um den WBA verstärkt Feedback verfügbar zu machen, wurden in der Anästhesiologie an einem Universitätsklinikum rund um Arbeitsplatzrotationen Feedbackgespräche (FBG) mit Lehrärzt:innen eingeführt. Die Effekte der FBG wurden mit Umfragen vor und nach der Einführung zu Feedback und Mitarbeiterzufriedenheit unter den WBA untersucht.

## Hintergrund und Fragestellung

„Feedback ist einer der stärksten Einflussfaktoren auf Lernen und Leistung“ [6], denn die Überwachung des Lernprozesses sowie das Verfügbarmachen von Feedback stellen einen der größten Stimuli für einen effektiven Lernfortschritt dar [5].

Feedback im klinischen Lernumfeld soll zeitgerecht und regelmäßig gegeben werden, in neutraler Sprache und in einem wertschätzenden und respektvollen Lern- bzw. Arbeitsklima [12]. Es sollen Reflexionen gefördert, Lernziele und konkrete Beobachtungen besprochen sowie ein Ausblick erarbeitet werden [12].

Schon vor langer Zeit wurden sowohl die Forderung für mehr FBG im Allgemeinen [14] als auch konkreter für Weiterbildungsgespräche (WBG) mit Zielvereinbarungen, die als für die Weiterbildung erfolgsrelevant bezeichnet wurden, und mit Rückmeldungscharakter über Leistungsfeedback beschrieben [16]. Der Mangel an individuellem und speziellem Feedback über Stärken und Schwächen [10] wurde zudem kritisiert. Obwohl mittlerweile durch die Weiterbildungsordnungen der Landesärztekammern (§ 8 (1) im Abschnitt A – Paragraphenteil) für die Qualifikation als Fachärztin/Facharzt jährliche WBG vorgesehen sind [2, 3, 16], wurden diese einerseits nicht an allen Kliniken regelmäßig durchgeführt [1, 3]. Andererseits schien trotz stellenweise etablierter WBG [15] ein subjektives Gefühl an mangelnder klinischer Rückmeldung weiterhin zu bestehen. Die WBA in dieser Klinik äußerten Unzufriedenheit über Quantität und Qualität von Feedback im klinischen Alltag. Dies deckt sich mit Erfahrungen andernorts, wo 80 % der WBA berichten, nie oder unregelmäßig Feedback von den Lehrärzt:innen zu erhalten [9].

Um daher die klinische Weiterbildung in der Klinik für Anästhesiologie am Universitätsklinikum Hamburg-Eppendorf (UKE) durch eine Steigerung an verfügbar gemachtem Feedback weiterzuentwickeln, wurden regelmäßige strukturierte, klinische FBG in Verbindung mit den klinischen Arbeitsplatzrotationen etabliert.

Der Effekt der Einführung dieser FBG wurde unter den WBA zu allgemeiner Wahrnehmung von erhaltenem Feedback, etwaigem Wunsch nach mehr Feedback (klinischer Rückmeldung) und allgemeiner Zufriedenheit am Arbeitsplatz evaluiert.

Die Forschungsfrage war hierbei, ob sich durch die Einführung von regelmäßigen strukturierten, klinischen FBG Änderungen in der Zufriedenheit über Rückmeldung zur beruflichen Leistungsfähigkeit und zur Mitarbeiterzufriedenheit unter den WBA feststellen lassen.

## Studiendesign und Untersuchungsmethoden

In der Klinik und Poliklinik für Anästhesiologie am UKE wurde in einer monozentrischen Studie die Einführung von regelmäßigen FBG im Rahmen der klinischen Arbeitsplatzrotationen unter den anästhesiologischen WBA untersucht. Im Sinne einer „justification study“ wurde mittels je einer Umfrage vor und nach der Einführung der FBG ein Vorher-nachher-Vergleich durchgeführt.

Vor Beginn der ersten Umfrage teilte die Ethikkommission der Landesärztekammer Hamburg per Mail auf Anfrage mit, dass es sich bei dem geplanten Vorhaben ihrer Einschätzung nach um Ausbildungsforschung bzw. Qualitätssicherung und nicht um „Forschung am Menschen“ im engeren Sinne handelt, sodass ihrerseits keine Beratung durch die Ethik-Kommission als erforderlich angesehen wurde. Jeweils vor den Umfragen unter den Mitarbeitern wurde beim Wissenschaftlichen Personalrat des UKE die Zustimmung zur Durchführung eingeholt.

### Intervention: Einführung der FBG

Als Intervention wurden mit Beginn des Jahres 2018 regelmäßige Lehrgespräche durch Lehrärzt:innen mit den WBA als sogenannte FBG eingeführt. Diese Gespräche sollten zu Beginn (Einführungsgespräch) und zum Ende (Abschlussgespräch) von zwei- bis dreimonatigen Arbeitsplatzrotationen in den verschiedenen OP-Bereichen (auch intern „Cluster“ genannt: OP-Bereiche mit vier „geclusterten“ OP) abgehalten werden. Bei dreimonatigen Rotationen war ein zusätzliches FBG in der Mitte des Arbeitsplatzeinsatzes („Halbzeit“-Gespräch) vorgesehen.

Ein speziell für diese FBG neu erstellter Dokumentationsbogen enthielt beispielhafte Fragen und Dokumentationsfelder, um als Hilfe zur Gesprächsstrukturierung zu dienen (Zusatzmaterial: Anhang A). In den Einführungsgesprächen sollte der Fokus auf die beiderseitigen Erwartungen gelegt werden („Was bringe ich mit?“). Das „Halbzeit“-Gespräch sollte im Falle zu einer Verlaufsbeurteilung eingesetzt werden („Wie läuft’s?“), um ggf. noch Korrekturen in der Ausbildung während der Rotation vornehmen bzw. Ausbildungsschwerpunkte ändern zu können. Das Abschlussgespräch sollte zum Fazit-Ziehen („Wie war’s?“) genutzt werden.

Für die Dauer der FBG war ein kurzer Zeitraum von 5–15 min vorgesehen, um die Befürchtung einer zu zeitintensiven Angelegenheit und damit Widerstände gegen die vorgesehene Durchführung zu entkräften. Als Lehrärzt:innen sollten Oberärzt:innen oder aufsichtsführende Fachärzt:innen (Fachärzt:innen mit Leitungsaufgaben) die FBG mit den WBA führen.

Auf dem Dokumentationsbogen waren auf der Rückseite für jedes der drei FBG 4 bis 6 Leitfragen formuliert, die als Hilfestellung zur Gesprächsstrukturierung gedacht waren. Auf der Vorderseite sollten die Ausbildungsziele formuliert werden. Für das Abschlussgespräch waren zudem inhaltliche Themen aufgelistet, deren Besprechung per Abhaken dokumentiert werden sollten. Lediglich sollte das Erreichen der zu Beginn festgelegten Ausbildungsziele und ggf. eine etwaige Begründung bei Nicht-Erreichen niedergeschrieben werden. Eine inhaltliche Bewertung sollte nicht erfolgen.

Die Durchführung der FBG wurde klinikintern als verpflichtend für den späteren Erwerb der Qualifikation als Fachärztin/Facharzt angesehen. Für den Nachweis der dokumentierten Gespräche sollten die WBA verantwortlich sein.

Im Januar 2018 wurde die Einführung dieser FBG per Mail angekündigt. Daneben diente im selben Monat eine 30-minütige klinikinterne Mitarbeiter:innenfortbildung zur Vorstellung des Projektes. Im Rahmen dieser Fortbildung wurden die Lehrärzt:innen und WBA auch darüber informiert, wie man Feedback geben:

- Ich-Botschaften,
- positive und negative Eindrücke,
- zeitnah,
- konkret und,
- fokussiert, wertfrei,

und Feedback nehmen;

- anhören und annehmen,
- nicht kommentieren und rechtfertigen,
- Unklarheiten klären, mit Rückmeldung beenden,

sollte.

### Umfrage vor der Intervention

Vor Einführung der FBG wurde im Mai 2017 eine freiwillige, anonyme und papierbasierte Umfrage unter den WBA (Zeitpunkt t_0_) zu 4 verschiedenen Themenbereichen mit insgesamt 28 einzelnen Items und Antwortmöglichkeiten auf einer 5‑teiligen Likert-Skala durchgeführt:

- Teil A (13 Items): allgemeine und spezielle Wahrnehmung von Feedback im OP-Einsatzbereich,
- Teil B (3 Items): subjektiver Wunsch bzw. Bedarf an Feedback im klinischen Bereich,
- Teil C (4 Items): Evaluation der jährlich stattfindenden WBG,
- Teil D (8 Items): allgemeine Zufriedenheit der Mitarbeiter zum Arbeitsplatz (Zusatzmaterial: Anhang B).

### Umfrage nach der Intervention

Etwa 14 Monate nach Einführung der FBG, im April 2019, wurde unter den WBA erneut eine freiwillige, anonyme und papierbasierte Umfrage (Zeitpunkt t_1_) unternommen. Diese enthielt 2 Anteile: Teil A, B und D (insgesamt 24 Items) wurden abermals abgefragt. Zusätzlich wurde diese Umfrage durch einen Teil E mit speziellen Evaluationsfragen (insgesamt 18 Items) zu den neu eingeführten FBG zu

- planmäßiger Durchführung,
- Struktur,
- Qualität und
- subjektiver Veränderung klinischer Rückmeldung

erweitert.

Verzichtet wurde auf die erneute Evaluation der WBG. Der Anteil des Gespräches um die klinische Entwicklung und Weiterbildung (Teil C) entfiel (Zusatzmaterial: Anhang C).

### Statistisches Vorgehen

Die Datensammlung erfolgte händisch durch die Übertragung der ausgefüllten und anonym und freiwillig abgegebenen papierbasierten Fragebogen in eine Tabellenkalkulationssoftware Microsoft Excel (Version 14.7.3, Microsoft Corporation, Redmond, WA, USA). Die statistische Auswertung wurde mit IBM SPSS Statistics (Version 29.0.0.0, IBM, Armonk, NY, USA) durchgeführt.

## Ergebnisse

An der ersten Umfrage zum Zeitpunkt t_0_ vor Einführung der FBG nahmen insgesamt 36 (20 weiblich, 16 männlich) von 71 WBA teil. Dies entsprach einer theoretischen Rücklaufquote von 51 %. Nach Einführung der FBG zum Zeitpunkt t_1_ beteiligten sich an der zweiten Umfrage 32 (18 weiblich, 15 männlich) von 73 WBA. Diese Rücklaufquote betrug damit 44 %.

### Evaluation der WBG

**Abb. 1:**
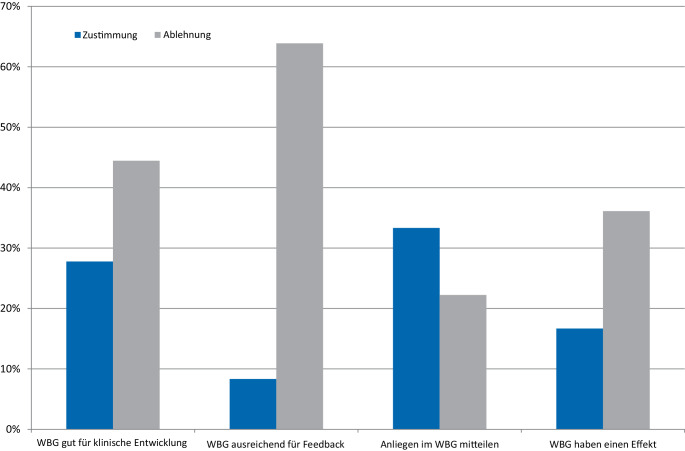
Deskriptive, prozentuale Darstellung (Teilnehmer *n* = 36 Weiterbildungsassistent:innen (WBA)) der Antworthäufigkeiten der Items zur Evaluation der Weiterbildungsgespräche (WBG) zum Zeitpunkt t_0_, zusammengefasst als Zustimmung („trifft voll zu“ oder „trifft eher zu“) oder Ablehnung („trifft eher nicht zu“ oder „trifft gar nicht zu“)

Zum Zeitpunkt t_0_ sprachen 44 % der Befragten („trifft eher nicht zu“ bzw. „trifft gar nicht zu“) den WBG einen Nutzen für die klinische Weiterentwicklung eher ab. 64 % beurteilten sogar, dass die WBG eher nicht bzw. nicht als Feedbackinstrument ausreichen würden (Abb. 1).

### Evaluation der FBG

Zum Zeitpunkt t_1_ wurde im Abschnitt E erst formal der Ablauf erfragt: Im Durchschnitt hätten theoretisch gemäß Arbeitsplatzrotationen 5,8 FBG pro WBA (insgesamt 169 FBG, im Median 6) stattfinden sollen. Tatsächlich wurden mit einem Anteil von 59,1 % im Mittel lediglich 3,4 FBG (insgesamt 100 FBG, im Median 2) pro WBA durchgeführt. Die mittlere Gesprächsdauer betrug 10,4 min (Median 10 min). Fast ausschließlich wurden diese Gespräche mit den Oberärzt:innen geführt; nur 13 von 100 FBG übernahmen die Fachärzt:innen mit Leitungsfunktion.

**Abb. 2:**
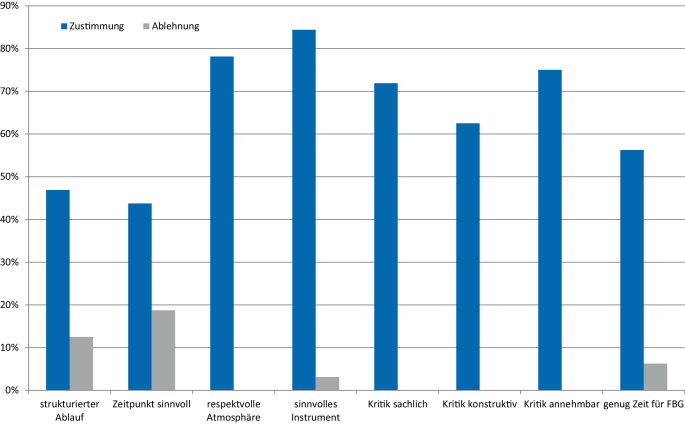
Deskriptive, prozentuale Darstellung (Teilnehmer *n* = 32 Weiterbildungsassistent:innen (WBA)) der Antworthäufigkeiten der Items zur Evaluation der Feedbackgespräche (FBG) zum Zeitpunkt t_1_, zusammengefasst als Zustimmung („trifft voll zu“ oder „trifft eher zu“) oder Ablehnung („trifft eher nicht zu“ oder „trifft gar nicht zu“)

In der subjektiven Beurteilung wurden von den Befragten den FBG in der fünf-teiligen Likert-Skala (im Folgenden Summe aus Antworten „trifft voll zu“ und „trifft eher zu“) attestiert, dass die FBG in wertschätzender und respektvoller Atmosphäre verliefen (78 %) und Kritik sachlich (72 %) und konstruktiv (63 %) geäußert wurde, so dass die Kritik großteils (75 %) als annehmbar wahrgenommen wurde (Abb. 2).

Bei der Befragung, ob sich durch die Einführung der FBG eine Veränderung von häufigeren Rückmeldungen, häufigerem nützlichen Rat und mehr Feedback zu Stärken und Schwächen ergeben haben, gab die unabhängige Stichprobe meist kein Urteil ab (50 %, 53 %, 56 % und 56 %). Allerdings wurden die FBG mit insgesamt 84 % Zustimmung allemal als sinnvolles Instrument angesehen.

### Vorher-nachher-Vergleich nach Einführung der FBG

Die abgegebenen Beurteilungen der jeweiligen WBA vor bzw. nach Einführung der FBG wurden itemweise mit t‑Tests für unabhängige Stichproben verglichen (zweiseitige Tests, Signifikanzschwelle *p* < 0,05). Eine Korrektur für multiples Testen wurde nicht vorgenommen; insofern sind die Ergebnisse trotz ihrer Signifikanz als deskriptiv zu betrachten.

Nach der Intervention zum Zeitpunkt t_1_ hatten die WBA eher den Eindruck, sie wüssten, wie ihre Vorgesetzten ihre praktischen Fähigkeiten einschätzten (Item 5; *p* = 0,017). So beurteilten die WBA zu t_1_ auch, ihren Vorgesetzten eher ihre eigenen Kritikpunkte und Verbesserungsvorschläge problemlos vortragen zu können (Item 13; *p* = 0,033). Zudem steigerte sich zu t_1_ unter den WBA das Gefühl, von den Vorgesetzten so behandelt zu werden, wie sie es sich wünschten (Item 26; *p* = 0,039).

**Abb. 3:**
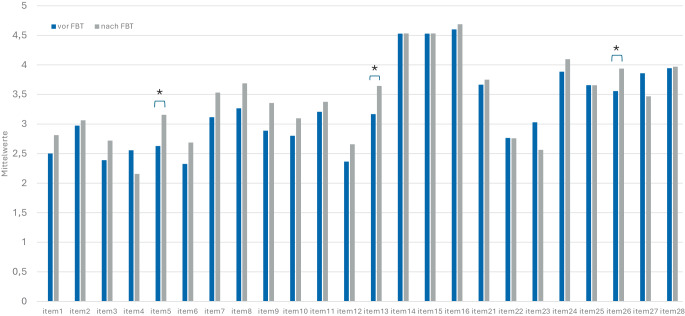
Vorher-nachher-Vergleich der wiederholt gefragten Items 1 bis 16 sowie 21 bis 28 aus der Umfrage zu Feedback und Mitarbeiterzufriedenheit unter den Weiterbildungsassistent:innen (WBA) zu Zeitpunkten t_o_ (*n* = 36) und t_1_ (*n* = 32) („trifft voll zu“ = 5 bis „trifft gar nicht zu“ = 1). Statistisch signifikante Änderungen ergaben sich in der mutmaßlichen Einschätzung der Vorgesetzten zu den praktischen Fähigkeiten der WBA (Item 5, *p* = 0,017), der Möglichkeit der WBA, eigene Anliegen den Vorgesetzten anbringen zu können (Item 13, *p* = 0,033), und in der gewünschten Behandlung der WBA durch die Vorgesetzten (Item 26, *p* = 0,039); (andere Items: Zusatzmaterial: Anhang B oder C)

Ein Trend zeigte sich in dem Eindruck, dass die WBA zum Zeitpunkt t_1_ eher die Rückmeldung erhalten, wo ihre Schwächen liegen (Item 4; *p* = 0,77), und dass sie besser beurteilen können, wie ihre Vorgesetzten ihre Teamfähigkeit (Item 7; *p* = 0,084) sowie den Umgang mit Patient:innen (Item 8; *p* = 0,07) und anderen Berufsgruppen (Item 9; *p* = 0,055) einschätzten. Demgegenüber wurden zu t_1_ grenzwertig signifikant weniger (Item 23; *p* = 0,05) die Angebote und Vorschläge zu klinischer und theoretischer Weiterbildung als ausreichend beurteilt (Abb. 3).

## Diskussion

Durch die Einführung von regelmäßigen strukturierten, klinischen FBG sind Hinweise zu erkennen, dass die Zufriedenheit über Rückmeldung zur beruflichen Leistungsfähigkeit und zur Feedbackkultur unter den WBA zunahm. Eine konkrete Steigerung der Arbeitsplatzzufriedenheit konnte nicht nachgewiesen werden.

Feedback stellt einen wichtigen Baustein für das Lernen dar. So fanden Hattie et al. heraus, dass das Verfügbarmachen von Feedback einen der größten Stimuli für einen effektiven Lernfortschritt bedeutet [5]. Schon 1983 wurde von Ende die wichtige Bedeutung von Feedback im klinischen Bereich beschrieben, dass ohne Feedback Fehler unkorrigiert bleiben, gute Leistungen nicht bestärkt werden und klinische Kompetenz empirisch oder überhaupt nicht erworben wird [4].

Auch Lernende messen Feedback eine große Bedeutung zu. Es beeinflusst maßgeblich die Zufriedenheit mit der Weiterbildung sowie dem Arbeitsplatz. Dies spielt damit eine Rolle je nach Sicht in der Wahl des Arbeitsplatzes oder eben in der Rekrutierung von Arbeitskräften.

Strukturierte Weiterbildungsprogramme könnten der mangelnden Zufriedenheit entgegenwirken [17]. Die Unzufriedenheit der WBA wird zu einem großen Teil auf das Fehlen von Feedback, Mentoring und strukturierter Anleitung während der Arbeitszeit als Merkmale einer qualitativ hochwertigen Weiterbildung zurückgeführt [10, 17]. Genannt werden dabei, dass fehlende Kritik am Arbeitsplatz ein Lob oder Gespräch über erlangte Ausbildungserrungenschaften nicht ersetzen könne. Strukturierte Interviews über Kompetenzen und mögliche Ausbildungsziele vor, während und nach Rotationen werden als hilfreich und sinnvoll angesehen [10].

In einer qualitativen Studie der European Junior Doctors von 2023 zeigten sich viele WBA enttäuscht und unzufrieden mit ihrer Arbeit. Beeinflusst würde dies sehr von der Qualität der Weiterbildung („lack of adequate supervision and feedback mechanisms“). Die medizinisch Lehrenden tragen Verantwortung an Letzterem und könnten maßgeblich dazu beitragen, die Gesamtsituation zu verbessern [7].

Siebolds betonte, ein wesentliches Unterscheidungsmerkmal von Kliniken im Wettbewerb um Assistenzärzte könnte ein gut dokumentiertes und evaluiertes Weiterbildungsprogramm sein. Diese Investitionen in die Weiterbildung von Ärzt:innen werde mitentscheidend für die Zukunfts- und Wettbewerbsfähigkeit von deutschen Kliniken und damit die Fachärzt:innenweiterbildung ein zentrales Instrument der Personalgewinnung sein [16].

Nach Einführung der FBG in unserer Studie wurde sich mehr gegenseitig Feedback gegeben und konnten häufiger Anliegen vorgebracht werden (*p* = 0,033). Eine größere Arbeitsplatzzufriedenheit war allerdings nicht zu erkennen.

Es existieren aber auch Widerstände gegen die Abhaltung von FBG. In Praxis und Klinik erschweren Zeitdruck und unerwartete Ereignisse ihre Durchführung [18]. Nach einer Onlinebefragung vom Marburger Bund von 2021 erhielten nur etwa 10 % der WBA regelmäßige Rückmeldungen und in 45 % nur einmal jährlich sowie 45 % gar nicht [18]. Auch Lägervik et al. beschrieben, die Weiterbildung ließe sich am meisten durch mehr Feedback verbessern, aber häufig wären Lehrärzt:innen aufgrund von hoher Arbeitsbelastung hierfür nicht verfügbar [11].

Um diesen Widerständen entgegenzuwirken, sollten die FBG in unserer Intervention bewusst auf 5–15 min beschränkt werden, was sich auch mit einer Dauer von 10,4 min im Mittel umsetzen ließ.

Trotz der Forderung, Feedback in einer strukturierten Form in die ärztliche Weiterbildung zu integrieren [10, 14, 16], können dies die von den ärztlichen Weiterbildungen vorgeschriebenen WBG [2] nur unzureichend leisten [1, 3]. Es gibt Hinweise, dass das Timing für den Effekt des „Multisource Feedback“ alle 3 bis 6 Monate wirksamer als einmal jährlich wäre [8]. Die einmal im Jahr vorgeschriebenen WBG könnten daher nur eine geringere Wirkung auf den Lernfortschritt erzielen. In unserer Studie wurde die Frequenz von einem WBG pro Jahr theoretisch auf 5,8 pro Jahr erhöht, doch tatsächlich fanden durchschnittlich nur 3,4 FBG statt.

In einer bundesweiten Befragung zur Weiterbildung durch Aulenkamp et al. fanden trotz des mittlerweile für die Weiterbildung verpflichtenden Charakters die WBG [2] nur bei etwas mehr als der Hälfte (51,7 %) der WBA regelmäßig, bei 37,2 % unregelmäßig und bei 11,0 % gar nicht statt. Die Mehrheit der Befragten war mit der Qualität der Weiterbildungsgespräche unzufrieden (61,2 %) und hatte den Eindruck, dass sie die Qualität der Weiterbildung nicht verbessern würden [1]. Ähnlich gaben in unserer Studie 64 % der Befragten an, mit den stabil einmal jährlich stattfindenden WBG als Feedbackinstrument unzufrieden zu sein.

Aulenkamp et al. formulierten die Forderung, „in Zukunft klare strukturelle Vorgaben für den Gesprächsablauf“ machen zu lassen [1]. Obwohl Supervision durch Oberärzt:innen und Fachärzt:innen als das wertvollste Instrument der Weiterbildung und FB zu 41,1 % als „besonders hilfreiches Instrument“ von den befragten WBA angesehen würden, sei FB weiterhin kein großes Thema in der Weiterbildung [1]. In unserer Untersuchung bescheinigten nach Einführung der FBG sogar 84 % der Befragten den FGB, ein „sinnvolles Instrument für die klinische Weiterentwicklung“ zu sein.

Um das Verfügbarmachen von Feedback und die Implementierung von FBG zu unterstützen, wird der Ruf nach einer Feedbackkultur im klinischen Umfeld laut. „Machen Sie Feedback zum Teil Ihrer Unternehmenskultur“, wird von Ramani et al. gefordert [12]. Eine Feedbackkultur ist auch wichtig für ein bidirektionales Feedback [13].

Fabry et al. sprechen sich ebenso für eine Lern- und Feedbackkultur aus, in der Feedback regelmäßig nach hinreichender Beobachtung der Lehrenden erfolgt, und die eine stabile, respektvolle und unterstützende Beziehung zwischen Lehrenden und Lernenden fördere [19]. Unsere Einführung der FBG führte erfolgreich vermehrt zu einem gefühlten Wissen unter den WBA, wie ihre Vorgesetzten ihre praktischen Fähigkeiten beurteilten (*p* = 0,017). Auch beurteilten die WBA eher, von den Vorgesetzten so behandelt zu werden, wie sie es wünschten (*p* = 0,039). Diese Bewertung und auch die Einschätzung unter den Befragten nach Einführung der FBG, dass in diesen Kritik sachlich (72 %), konstruktiv (63 %) und gut annehmbar (75 %) geäußert werden konnten und die FBG in wertschätzender und respektvoller Atmosphäre (78 %) ablaufen würden, wird als Zeichen für den Erfolg der neu initiierten FBG in Bezug auf Feedbackkultur gewertet.

Feedback sollte in Lehr-Lern-Prozesse eingebettet sein, Ziele transparent kommuniziert und mit einem Aktion- und Handlungsplan verknüpft sein („Welche Ziele sollen erreicht werden? Wie geschieht dies momentan, und wie kann und soll es weitergehen?“) [19]. Diese Forderungen unterstützen unser Vorgehen, einerseits die FBG im Rahmen der Arbeitsplatzrotationen strukturell sinnvoll in die klinische Weiterbildung eingepflegt zu haben („curriculum alignment“) und andererseits mit den auf dem Dokumentationsbogen formulierten Leitfragen eine Hilfestellung zur Gesprächsstrukturierung geliefert zu haben.

### Limitationen

In unserer monozentrischen Untersuchung blieben die Stichproben aufgrund der Personalstärke der Klinik trotz passabler Rücklaufquote von etwa der Hälfte der Zielgruppe klein. Die Untersuchung war nur mit unabhängigen, also nicht personenidentischen Stichproben durchführbar. Befragt wurde auf dem Level der subjektiven Wahrnehmung mit selbsterstellten und nicht wissenschaftlich validierten Umfrage-Items.

Bezüglich der Projektplanung wäre eine intensivere Schulung der Lehrärzt:innen in Feedback-Geben und Personalgesprächsführung nicht nachteilig gewesen. Zudem gab es keine engmaschige Kontrolle für die Durchführung der FBG, sodass der Anteil von knapp 60 % durchgeführten an theoretisch vorgesehenen FBG noch als steigerbar angesehen werden dürfte.

## Fazit für die Praxis

- Feedback zu erhalten, stellt einen großen Stimulator für den Lernprozess dar.
- Subjektiv und objektiv erhalten Weiterbildungsassistent:innen (WBA) zu wenig klinisches Feedback in der Weiterbildung.
- Von den Weiterbildungsordnungen verpflichtende Weiterbildungsgespräche (WBG) als Feedbackinstrument finden nicht überall zuverlässig statt. Sie werden als zu selten angesehen und kritisch betrachtet.
- Mehrmalige Feedbackgespräche (FBG) pro Jahr werden von den WBA überwiegend begrüßt und als sinnvolles Feedbackinstrument erachtet.
- Die FBG ermöglichen ein Forum, in dem WBA auch Rückmeldungen an die Verantwortlichen der Klinik geben können.
- Die FBG scheinen einen Beitrag für eine Feedbackkultur und wertschätzendes und respektvolles Arbeitsklima zu leisten.

## Supplementary Information

ESM1: Zusatzmaterial 1

## Data Availability

Die in dieser Studie erhobenen Datensätze können auf begründete Anfrage beim Korrespondenzautor angefordert werden.

## References

[1] Aulenkamp J, Guddat C, Krug N (2023) Evaluation der Arbeitsund Weiterbildungsbedingungen im Fachgebiet Anästhesiologie in Deutschland 2021 (Ergebnisse einer online-basierten Befragung). Anaesthesiologie & Intensivmedizin 64

[2] Bundesaerztekammer (2018) (Muster‑)Weiterbildungsordnung (MWBO) vom 15.11.2018. https://www.bundesaerztekammer.de/themen/aerzte/aus-fort-und-weiterbildung/aerztliche-weiterbildung/muster-weiterbildungsordnung

[3] Bussche H, Krause-Solberg L, Scherer M et al (2017) Lernprozesse und Lernprobleme in der ärztlichen Weiterbildung in Deutschland. GMS J Med Educ 34:Doc 54

[4] Ende J (1983) Feedback in clinical medical education. Jama 250:777–781 6876333

[5] Hattie J, Beywl W, Zierer K (2013) Lernen sichtbar machen. Schneider-Verl, Hohengehren

[6] Hattie J, Timperley H (2007) The power of feedback. Review of educational research 77:81–112

[7] Hennel EK, Fehr F, Wijnen-Meijer M et al (2024) Postgraduate medical education in change: A long journey. GMS Journal for Medical Education 41:Doc 69 10.3205/zma001724 PMC11656170 39711867

[8] Hennel EK, Trachsel A, Subotic U et al (2022) How does multisource feedback influence residency training? A qualitative case study. Medical education 56:660–669 35263461 10.1111/medu.14798 PMC9314722

[9] Hewson MG, Little ML (1998) Giving feedback in medical education: verification of recommended techniques. Journal of general internal medicine 13:111–116 9502371 10.1046/j.1525-1497.1998.00027.x PMC1496906

[10] Iblher P, Hofmann M, Zupanic M et al (2015) What motivates young physicians?–a qualitative analysis of the learning climate in specialist medical training. BMC medical education 15:1–7 26471718 10.1186/s12909-015-0461-8 PMC4608325

[11] Lägervik M, Thörne K, Fristedt S et al (2022) Residents’ and supervisors’ experiences when using a feedback-model in post-graduate medical education. BMC Medical Education 22:891 36564770 10.1186/s12909-022-03969-5 PMC9789576

[12] Ramani S, Krackov SK (2012) Twelve tips for giving feedback effectively in the clinical environment. Medical teacher 34:787–791 22730899 10.3109/0142159X.2012.684916

[13] Ramani S, Post SE, Könings K et al (2017) „It’s just not the culture“: a qualitative study exploring residents’ perceptions of the impact of institutional culture on feedback. Teaching and learning in medicine 29:153–161 28001442 10.1080/10401334.2016.1244014

[14] Schaaf W, Zwißler B (2012) Evaluation der Weiterbildung in der Anästhesie. Anaesthesist 61 10.1007/s00101-012-2073-6 22941388

[15] Schmidt G, Fiege M, Goetz A (2011) Weiterbildung in der Anästhesiologie. Anaesthesist 60 10.1007/s00101-010-1836-1 21479708

[16] Siebolds M, Beer AM, Kiwitt P et al (2006) Alter Wein in neuen Schläuchen oder Zukunftsoption? Dtsch Ärztebl 103:A2765–A2768 18062035

[17] Sierocinski E, Mathias L, Pereira JFM et al (2022) Postgraduate medical training in Germany: A narrative review. GMS Journal for Medical Education 39:Doc 49 10.3205/zma001570 PMC9733474 36540556

[18] Spielberg P (2023) Dialogkultur auf Augenhöhe. Dtsch Ärztebl 120:A310–A311

[19] Thrien C, Fabry G, Härtl A et al (2020) Feedback in medical education–a workshop report with practical examples and recommendations. GMS journal for medical education 37:Doc 46 10.3205/zma001339 PMC7499466 32984505

