# Supplementary material for: Feedback conversations in postgraduate medical education: Structured feedback in anesthesiological postgraduate education at the University Medical Center Hamburg-Eppendorf —A pre-post analysis
Source: Anaesthesiologie. 2026 Jun 24;75(8):549–56. [Article in German] doi: 10.1007/s00101-026-01700-3 (PMC13427792; doi:10.1007/s00101-026-01700-3)
Supplement: Supplementary file 1 — ESM1: Zusatzmaterial 1 [file 101_2026_1700_MOESM1_ESM.pdf]

Zusatzmaterial zum Beitrag „Feedbackgespräche in der Weiterbildung. Strukturiertes Feedback in der anästhesiologischen Weiterbildung am UKE - eine Prä-Post-Analyse“ von Nicolas Hoffmann, Martin Klasen, Katharina Röher, Saša Sopka (2026) in *Die Anaesthesiologie.*

Beitrag und Zusatzmaterial stehen Ihnen auf www.springermedizin.de zur Verfügung. Bitte geben Sie dort den Beitragstitel in die Suche ein.

Feedbackgespräche

Name des Weiterbildungsassistenten: ………………................ Cluster / OP-Bereich:……….

Einführungsgespräch (zu Beginn der Cluster-Rotation): Datum:...................

Formulierte(s) Ziel(e) für dieses Cluster:

……………………………………………………………………………………………………

……………………………………………………………………………………………………

Unterschrift Weiterbildungsassistent Unterschrift Facharzt 1.Dienst / Oberarzt

---

„Halbzeit“-Gespräch (ca. nach 4-6 Wochen): Datum:...................

Was muss zur Erreichung der formulierten Ziele noch verbessert werden?

……………………………………………………………………………………………………

Unterschrift Weiterbildungsassistent Unterschrift Facharzt 1.Dienst / Oberarzt

---

Abschlussgespräch (am Ende der Cluster-Rotation): Datum:...................

Es sollten über folgende Themen in einem strukturiertem Feedback-Gespräch gesprochen werden (bitte abhaken):

O Gesamteindruck des Assistenzarztes

O Teamfähigkeit

O Umgang mit Patienten, Pflegepersonal, ärztlichen Kollegen, anderen Fachdisziplinen

O Verhalten in Routinesituationen

O Verhalten bei unerwarteten anästhesiologischen und operativen Problemen

O Durchführung praktischer Fertigkeiten

O Theoretische Kenntnisse

O Feedback an den Bereichsleiter, Zufriedenheit mit der Rotation

Wurde das formulierte Ziel des Einführungsgespräches erreicht? Wenn nein, warum nicht?

……………………………………………………………………………………………………

……………………………………………………………………………………………………

……………………………………………………………………………………………………

Unterschrift Weiterbildungsassistent Unterschrift Facharzt 1.Dienst / Oberarzt

© Hoffmann, Röher

Hinweise zu den Feedbackgesprächen:

Zu Beginn einer Rotation im Rahmen der ärztlichen Weiterbildung in unserer Klinik übernimmt ein Facharzt 1.Dienst / Oberarzt des Arbeitsbereiches die Rolle des Lehrarztes die Aufgabe, Sie in der Weiterbildung anhand dieser Gespräche zu unterstützen. Er soll Ihnen als kollegialer Ansprechpartner für Fragen und Probleme während der Ausbildung zur Verfügung stehen. Der Lehrarzt führt regelmäßig mit Ihnen Gespräche zum Ausbildungsverlauf durch.

Einführungsgespräch: ***Was bringe ich mit? Was erwarte ich?***

Jeweils zu Beginn einer Rotation findet ein ca. 5-15 minütiges Einführungsgespräch statt. Neben einer Selbsteinschätzung und Erwartungen sollen persönliche Ziele für den kommenden Ausbildungsabschnitt besprochen werden.

Folgende Leitfragen sollen Sie für das Gespräch unterstützen:

- Welche klinischen Vorerfahrungen habe ich bereits gemacht (Berufsanfänger mit speziellen Praktika/Famulaturen, andere Rotationen bereits absolviert)?
- Welche Fähigkeiten/Fertigkeiten/Stärke bringe ich bereits mit?
- Wo sehe ich bei mir noch Verbesserungsmöglichkeiten/Schwächen?
- Was sind meine persönlichen Ziele? Was möchte ich unbedingt lernen?

„Halbzeit"-Gespräch: ***Wie läuft´s? Was habe ich erreicht? Was fehlt?***

Jeweils nach 4-6 Wochen der Rotation ein ca. 5-10 minütiges Verlaufsgespräch. Neben einer erneuten Selbsteinschätzung zum Lernfortschritt sollen bei Bedarf Probleme und Ausbildungsdefizite angesprochen werden. Der Lehrarzt gibt Ihnen ein strukturiertes Feedback zu Ihrer persönlichen Entwicklung (Stärken, Verbesserungspotential, Ausblick).

Folgende Leitfragen sollen Sie für das Gespräch unterstützen:

- Was waren die Stärken im bisherigen Verlauf der Rotation?
- Was habe ich insbesondere gelernt (theoretisch/praktisch)?
- Wie bin ich in das Team integriert (Bereichsleitung? Kollegen? Pflege? Operateure?)?
- Was waren die Schwachstellen im bisherigen Verlauf des Tertials/Rotation?
- Wo habe ich persönlich noch Nachholbedarf? Was bringe ich selbst ein?
- Welche Lernangebote brauche ich noch?

Abschlussgespräche: ***Wie war´s? Habe ich meine Ziele erreicht? Bin ich zufrieden?***

Jeweils am Ende einer Rotation findet ein ca. 10-15 minütiges Abschlussgespräch statt. Neben einer erneuten Selbsteinschätzung Ihrer Entwicklung und Erreichen Ihre Lernziele können Sie Kritik und Verbesserungsvorschläge zu dem Weiterbildungsabschnitt äußern. Der Lehrarzt gibt Ihnen erneut ein strukturiertes Feedback zu Ihrer persönlichen Entwicklung (Stärken, Verbesserungspotential, Ausblick).

Folgende Leitfragen sollen Sie für das Gespräch unterstützen:

- Was waren die positiven Aspekte/Highlights der Rotation?
- Was habe ich insbesondere gelernt (theoretisch/praktisch)?
- Wie war ich in das Team integriert(Bereichsleitung? Kollegen? Pflege? Operateure?)?
- Was waren die Schwachstellen/Verbesserungspotential der Rotation?
- Welche Ziele konnte ich nicht erreichen?
- Welche Verbesserungsvorschläge gibt es für die künftige Ausbildung?

Fragebogen *Untersuchung "Feedback über klinische Tätigkeit"*

Datum: ____

Pseudonymisierung Assistentsarzt/in (bitte ausfüllen)
(dadurch wird Zuordnung ggf. bei weiterer Befragung möglich)

erste 3 Buchstaben des Vornamens der Mutter	erste 3 Buchstaben des Vornamens des Vaters	eigener Geburtsmonat

Biografische Daten Assistentsarzt/in (bitte ausfüllen)

Geschlecht	Alter	aktuelle Dienstgruppe

Wahrnehmung Feedback im OP-Bereich / Cluster:

In diesem Abschnitt interessiert uns Ihre konkrete Wahrnehmung von Feedback in den einzelnen OP-Bereichen. Bitte folgende Fragen/Items gemäß folgender Skala ankreuzen (bitte jeweils nur ein Kreuz pro Item):

		trifft voll zu	trifft eher zu	teils teils	trifft eher nicht zu	trifft gar nicht zu	weiß nicht	kann ich nicht beurteilen
Allgemein:								
	**Ich erhalte...**							
1	...ausreichend häufig Rückmeldung über meine klinische Tätigkeit in der Rotation.	o	o	o	o	o	o	o
2	...angemessene, nützliche Ratschläge, um mich zeitnah klinisch weiterzuentwickeln.	o	o	o	o	o	o	o
3	...Rückmeldung, wo Stärken meiner klinischen Arbeit liegen.	o	o	o	o	o	o	o
4	...Rückmeldung, wo Schwächen meiner klinischen Arbeit liegen.	o	o	o	o	o	o	o
Speziell:								
	**Ich weiß, wie meine Vorgesetzten ...**							
5	...meine praktischen Fertigkeiten einschätzen.	o	o	o	o	o	o	o
6	...meine theoretischen Kenntnisse einschätzen.	o	o	o	o	o	o	o
7	...meine Teamfähigkeit einschätzen.	o	o	o	o	o	o	o
8	...meinen Umgang mit Patienten einschätzen.	o	o	o	o	o	o	o
9	...meinen Umgang mit anderen Berufsgruppen einschätzen.	o	o	o	o	o	o	o
10	...meinen Umgang mit anderen Fachdisziplinen einschätzen.	o	o	o	o	o	o	o
11	...mein Verhalten in Routinesituationen einschätzen.	o	o	o	o	o	o	o
12	...mein Verhalten in Notfallsituationen einschätzen.	o	o	o	o	o	o	o
Rückmeldung:								
13	Ich kann meinem Vorgesetzten meine Kritikpunkte und Verbesserungsvorschläge problemlos vortragen.	o	o	o	o	o	o	o

Bitte ausgefüllten Bogen in den Briefumschlag legen, verschließen und in das gekennzeichnete Fach legen.
Vielen Dank für Ihre Mitarbeit!

In den folgenden Abschnitten bitte die jeweiligen Fragen/Items ebenfalls gemäß folgender Skala ankreuzen (bitte jeweils nur ein Kreuz pro Item):		trifft voll zu	trifft eher zu	teils teils	trifft eher nicht zu	trifft gar nicht zu	weiß nicht	kann ich nicht beurteilen
**Wunsch / Bedarf für Feedback im OP-Bereich / Cluster:**								
14	Ich halte regelmäßige Feedbackgespräche in ruhiger Atmosphäre für sinnvoll.	o	o	o	o	o	o	o
15	Ich wünsche mir mehr unmittelbares Feedback in konkreten klinischen Situationen zu meiner Tätigkeit.	o	o	o	o	o	o	o
16	Ich wünsche mir strukturierte Rückmeldungen zu speziellen Tätigkeiten, um diese in Zukunft eigenständig durchführen zu können.	o	o	o	o	o	o	o
**Wahrnehmung Weiterbildungsgespräche (als Teil der Mitarbeiter-Jahresgespräche) bezüglich klinischer Tätigkeit:**								
	**Die Rückmeldung über meine klinische Tätigkeit in den Weiterbildungsgesprächen (als Teil der Mitarbeiter-Jahresgespräche) ...**							
17	...tragen sehr zu meiner klinischen Entwicklung bei.	o	o	o	o	o	o	o
18	...empfinde ich als Feedbackinstrument ausreichend.	o	o	o	o	o	o	o
19	Ich kann meine Anliegen über meine klinische Tätigkeit ausreichend in den Weiterbildungsgesprächen (als Teil der Mitarbeiter-Jahresgespräche) zum Ausdruck bringen.	o	o	o	o	o	o	o
20	Meine klinischen Anliegen, die ich in den Weiterbildungsgesprächen (als Teil der Mitarbeiter-Jahresgespräche) anspreche, werden zufriedenstellend behandelt.	o	o	o	o	o	o	o
**Mitarbeiterzufriedenheit allgemein**								
21	Es fällt schwer, sich in neue Bereiche einzuarbeiten.	o	o	o	o	o	o	o
22	Ich kann Wünsche bezüglich meiner klinischen Weiterbildung einbringen.	o	o	o	o	o	o	o
23	Angebote und Vorschläge zu klinischer und theoretischer Weiterbildung sind ausreichend.	o	o	o	o	o	o	o
24	Ich werde von meinen Vorgesetzten ernst genommen.	o	o	o	o	o	o	o
25	Kritik wird sachlich und konstruktiv geäussert.	o	o	o	o	o	o	o
26	Ich werde von meinen Vorgesetzten so behandelt, wie ich es mir wünsche.	o	o	o	o	o	o	o
27	Ich würde unsere Klinik als Weiterbildungsstätte anderen Ärzten weiterempfehlen.	o	o	o	o	o	o	o
28	Ich bin mit meinem Arbeitsplatz zufrieden.	o	o	o	o	o	o	o

Bitte ausgefüllten Bogen in den Briefumschlag legen, verschließen und in das gekennzeichnete Fach legen.
Vielen Dank für Ihre Mitarbeit!

Fragebogen *Untersuchung "Feedback über klinische Tätigkeit"*

März/April 2019

Biografische Daten Assistenzärztin oder -arzt (bitte markieren)

m / w	2. / 3. / 4.
Geschlecht	aktuelle Dienstgruppe

A) Wahrnehmung Feedback im OP-Bereich / Cluster:

In diesem Abschnitt interessiert uns Ihre konkrete Wahrnehmung von Feedback in den einzelnen OP-Bereichen. Bitte folgende Fragen/Items gemäß folgender Skala ankreuzen (bitte jeweils nur ein Kreuz pro Item):

		trifft voll zu	trifft eher zu	teils teils	trifft eher nicht zu	trifft gar nicht zu	weiß nicht	kann ich nicht beurteilen
Allgemein:								
	**Ich erhalte...**							
1	...ausreichend häufig Rückmeldung über meine klinische Tätigkeit in der Rotation.	o	o	o	o	o	o	o
2	...angemessene, nützliche Ratschläge, um mich zeitnah klinisch weiterzuentwickeln.	o	o	o	o	o	o	o
3	...Rückmeldung, wo Stärken meiner klinischen Arbeit liegen.	o	o	o	o	o	o	o
4	...Rückmeldung, wo Schwächen meiner klinischen Arbeit liegen.	o	o	o	o	o	o	o
Speziell:								
	**Ich weiß, wie meine Vorgesetzten ...**							
5	...meine praktischen Fertigkeiten einschätzen.	o	o	o	o	o	o	o
6	...meine theoretischen Kenntnisse einschätzen.	o	o	o	o	o	o	o
7	...meine Teamfähigkeit einschätzen.	o	o	o	o	o	o	o
8	...meinen Umgang mit Patienten einschätzen.	o	o	o	o	o	o	o
9	...meinen Umgang mit anderen Berufsgruppen einschätzen.	o	o	o	o	o	o	o
10	...meinen Umgang mit anderen Fachdisziplinen einschätzen.	o	o	o	o	o	o	o
11	...mein Verhalten in Routinesituationen einschätzen.	o	o	o	o	o	o	o
12	...mein Verhalten in Notfallsituationen einschätzen.	o	o	o	o	o	o	o
Rückmeldung:								
13	Ich kann meinem Vorgesetzten meine Kritikpunkte und Verbesserungsvorschläge problemlos vortragen.	o	o	o	o	o	o	o

Bitte ausgefüllten Bogen in den Briefumschlag legen, verschließen und in das gekennzeichnete Fach legen.
Vielen Dank für Ihre Mitarbeit!

Nr.	In den folgenden Abschnitten bitte die jeweiligen Fragen/Items ebenfalls gemäß folgender Skala ankreuzen (bitte jeweils nur ein Kreuz pro Item):	trifft voll zu	trifft eher zu	teils teils	trifft eher nicht zu	trifft gar nicht zu	weiß nicht	kann ich nicht beurteilen
**B)**	**Wunsch / Bedarf für Feedback im OP-Bereich / Cluster:**							
14	Ich halte regelmäßige Feedbackgespräche in ruhiger Atmosphäre für sinnvoll.	o	o	o	o	o	o	o
15	Ich wünsche mir mehr unmittelbares Feedback in konkreten klinischen Situationen zu meiner Tätigkeit.	o	o	o	o	o	o	o
16	Ich wünsche mir strukturierte Rückmeldungen zu speziellen Tätigkeiten, um diese in Zukunft eigenständig durchführen zu können.	o	o	o	o	o	o	o
**D)**	**Mitarbeiterzufriedenheit allgemein**							
21	Es fällt schwer, sich in neue Bereiche einzuarbeiten.	o	o	o	o	o	o	o
22	Ich kann Wünsche bezüglich meiner klinischen Weiterbildung einbringen.	o	o	o	o	o	o	o
23	Angebote und Vorschläge zu klinischer und theoretischer Weiterbildung sind ausreichend.	o	o	o	o	o	o	o
24	Ich werde von meinen Vorgesetzten ernst genommen.	o	o	o	o	o	o	o
25	Kritik wird sachlich und konstruktiv geäussert.	o	o	o	o	o	o	o
26	Ich werde von meinen Vorgesetzten so behandelt, wie ich es mir wünsche.	o	o	o	o	o	o	o
27	Ich würde unsere Klinik als Weiterbildungsstätte anderen Ärzten weiterempfehlen.	o	o	o	o	o	o	o
28	Ich bin mit meinem Arbeitsplatz zufrieden.	o	o	o	o	o	o	o

E) Zusätzliche Evaluationsfragen nach Einführung Feedbackgespräche im Cluster/OP-Bereich (t_1)

29 Wie viele Feedbackgespräche waren im vergangenen Jahr 2018 theoretisch für mich vorgesehen? *(Summe aus: 3 Gespräche pro 3-Monats-Rotation, 2 Gespräche pro 4- bzw. 8-Wochen-Rotation)* ☐

30 Wie viele dieser vorgesehenen Feedbackgespräche haben tatsächlich stattgefunden? ☐

31 Wie viele Gespräche wurden einem Facharzt/in 1.Dienst (und **nicht** von einem Oberarzt/in) geführt? ☐

32 Wie lange (in Minuten) dauerte etwa im Durchschnitt ein Feedbackgespräch? ☐

Bitte ausgefüllten Bogen in den Briefumschlag legen, verschließen und in das gekennzeichnete Fach legen.
Vielen Dank für Ihre Mitarbeit!

Nr.	In den folgenden Abschnitten bitte die jeweiligen Fragen/Items ebenfalls gemäß folgender Skala ankreuzen (bitte jeweils nur ein Kreuz pro Item):	trifft voll zu	trifft eher zu	teils teils	trifft eher nicht zu	trifft gar nicht zu	weiß nicht	kann ich nicht beurteilen
	**Die Feedbackgespräche im Cluster/OP-Bereich...**							
33	...verlaufen strukturiert ab.	o	o	o	o	o	o	o
34	...verlaufen zu sinnvollen Zeitpunkten ab.	o	o	o	o	o	o	o
35	...verlaufen in wertschätzender und respektvoller Atmosphäre.	o	o	o	o	o	o	o
36	...wurden von den Oberarzt/innen initiiert.	o	o	o	o	o	o	o
37	...wurden von mir als Weiterbildungsassistent/in initiiert.	o	o	o	o	o	o	o
38	...halte ich für ein sinnvolles Instrument für meine klinische Weiterentwicklung.	o	o	o	o	o	o	o
	**In den Feedbackgesprächen im Cluster/OP-Bereich...**							
39	...wird Kritik sachlich geäussert.	o	o	o	o	o	o	o
40	...wird Kritik konstruktiv geäussert.	o	o	o	o	o	o	o
41	...kann ich geäußerte Kritik gut annehmen.	o	o	o	o	o	o	o
42	...war ausreichend Zeit vorhanden.	o	o	o	o	o	o	o
	**Seit der Einführung der Feedbackgespräche erhalte ich...**	o	o	o	o	o	o	o
43	...**häufiger** Rückmeldung über meine klinische Tätigkeit in der Rotation.	o	o	o	o	o	o	o
44	...**mehr** angemessene, nützliche Ratschläge, um mich zeitnah klinisch weiterzuentwickeln.	o	o	o	o	o	o	o
45	...**mehr** Rückmeldung, wo Stärken meiner klinischen Arbeit liegen.	o	o	o	o	o	o	o
46	...**mehr** Rückmeldung, wo Schwächen meiner klinischen Arbeit liegen.	o	o	o	o	o	o	o

Bitte ausgefüllten Bogen in den Briefumschlag legen, verschließen und in das gekennzeichnete Fach legen.
Vielen Dank für Ihre Mitarbeit!
